# PGP: parallel prokaryotic proteogenomics pipeline for MPI clusters, high-throughput batch clusters and multicore workstations

**DOI:** 10.1093/bioinformatics/btu051

**Published:** 2014-01-27

**Authors:** Andrey Tovchigrechko, Pratap Venepally, Samuel H. Payne

**Affiliations:** ^1^J. Craig Venter Institute, 9704 Medical Center Drive, Rockville, MD 20850 and ^2^Pacific Northwest National Laboratory, 902 Battelle Blvd., Richland, WA 99354, USA

## Abstract

**Summary:** We present the first public release of our proteogenomic annotation pipeline. We have previously used our original unreleased implementation to improve the annotation of 46 diverse prokaryotic genomes by discovering novel genes, post-translational modifications and correcting the erroneous annotations by analyzing proteomic mass-spectrometry data.

This public version has been redesigned to run in a wide range of parallel Linux computing environments and provided with the automated configuration, build and testing facilities for easy deployment and portability.

**Availability and implementation:** Source code is freely available from https://bitbucket.org/andreyto/proteogenomics under GPL license. It is implemented in Python and C++. It bundles the Makeflow engine to execute the workflows.

**Contact:** atovtchi@jcvi.org

## 1 INTRODUCTION

Our pipeline is a tool for improving the existing genomic annotations from available proteomics mass spectrometry data. As most genome annotation pipelines consist of automated gene finding, they lack experimental validation of primary structure (Aziz *et al.*, 2008; Markowitz *et al.*, 2008), having to rely on DNA centric sources of data such as sequence homology, transcriptome mapping, codon frequency, etc. By incorporating the orthogonal set of data, proteogenomics is able to discover novel genes, post-translational modifications and correct the erroneous primary sequence annotations.

The protocol and the large-scale application of our original pipeline to 46 taxonomically diverse genomes were reported in Venter *et al.* (2011). The implementation was tightly coupled with the internal computation services framework (VICS) at the J. Craig Venter Institute (JCVI). VICS has never been deployed outside of the JCVI, and the pipeline itself required manual configuration and building by the developers. It could only use Sun Grid Engine (SGE) batch queuing system configured for high-throughput computing (HTC) mode in which large numbers of serial jobs could be efficiently scheduled on a compute cluster. For these reasons, the original pipeline has not been made public.

To create the first open source release presented here, we have redesigned the pipeline to run in a wide range of parallel Linux computing environments:

- High-performance computing (HPC) clusters, which are set up to efficiently schedule only large (100s+ of cores) parallel Message Passing Interface (MPI) jobs under a control of batch queuing system such as Sun Grid Engine (SGE) and its clones, Simple Linux Utility for Resource Management (SLURM) or Portable Batch System (PBS)/Torque. Our primary targets for this use case were compute clusters of XSEDE (https://www.xsede.org/), the federation of supercomputers supported by the US National Science Foundation. XSEDE allocates its resources to outside researchers through a peer-reviewed proposal system. The biologists will be able to use our software on this major computational resource.
- High-throughput computing (HTC) clusters widely used as local bioinformatics computing resources. These clusters are configured to efficiently schedule large numbers of serial jobs under a control of batch queuing system.
- A single multi-core workstation without a batch queuing system (including an extreme case of single-core machine).

The volume of computations in proteogenomics is relatively high, with ∼100 CPU hours for a typical bacterial genome. Our pipeline performs such annotation in ∼3 h of wall clock time on HTC cluster.

We have now designed a fully automated installation procedure preconfigured for several types of specific target systems and easily adaptable to others through editing of a few configuration files.

Although several other proteogenomic packages (Chapman *et al.*, 2013; Kumar *et al.*, 2013; Risk *et al.*, 2013; Sanders *et al.*, 2011) have been developed in recent years, they were designed for execution on a single workstation. None of the other publications matched the breadth of application reported for our pipeline in Venter *et al.* (2011).

The output files from that study are available at (http://omics.pnl.gov/pgp/overview.php). The contributed RefSeq updates can be seen in the Genbank flat files (.gbk) of the corresponding genomes at the NCBI wherever the proteomics data are listed as experimental evidence. One example is the *Mycobacterium tuberculosis* H37Rv genome (ftp://ftp.ncbi.nih.gov/genomes/Bacteria/Mycobacterium_tuberculosis_H37Rv_uid57777/NC_000962.gbk) containing the CDS attributes/experiment=“EXISTENCE: identified in proteomics study”.

## 2 ARCHITECTURE AND IMPLEMENTATION

### 2.1 Parallelization strategy

In the present work, our main goal was to make the same pipeline protocol portable across different parallel execution environments that users are likely to encounter. The original algorithm is embarrassingly parallel for the most part. It processes each spectrum file independently throughout all computationally intensive stages of the algorithm. There is a global synchronization point in the middle to build a histogram of all scores for *P*-value computation. Thus, the pipeline corresponds to a distributed workflow where multiple serial processes are executed concurrently following a dependency graph defined by required input and output files. This model is compatible with a wide variety of execution environments such as standalone multicore machines, HTC clusters and, with extra effort, MPI clusters. The original unreleased implementation used HTC model tied into VICS and SGE.

We have now achieved the portability across execution environments by generating and running the same workflow under the Makeflow engine (http://nd.edu/∼ccl/software/makeflow/) (Thrasher *et al.*, 2010) that provides parallel execution on multiple types of batch queuing systems as well as on standalone multicore nodes. On MPI clusters, Makeflow uses ‘glide-in’ approach that we describe in PGP software manual. In short, the ‘glide-in’ approach emulates an HTC cluster inside a single large MPI job.

It will be also trivial to deploy our pipeline behind any Web services front-end such as Galaxy (Giardine *et al.*, 2005) or Taverna (Wolstencroft *et al.*, 2013). Each run of the pipeline appears to the caller as a single command-line invocation of the entry point script that exits only once it finishes executing its parallel workflow. Backend options (batch queue or local multicore) are passed through the command arguments. No permanently running server components are used by Makeflow. Deployment in Galaxy, for example, would be the same as deployment of a simple serial tool, requiring creation of a single XML tool description file.

### 2.2 Installation and execution

Newly developed installation procedure and documentation are part of the source code repository. The step-by-step installation and usage manual (also shown on the landing page at BitBucket) covers the execution environments, specific examples of configuration files for each environment and instructions for adapting these files to new compute clusters.

The manual also covers sample run-time parameters for different environments, Quick Start instructions for testing the pipeline on a small dataset included in the repository and example of interpreting the pipeline’s output to discover a novel gene. The automated configuration and build procedure is driven by CMake (http://www.cmake.org/). Our installation procedure builds its own local copy of the Makeflow and several proteomics tools from (http://proteomics.ucsd.edu).

*Funding*: National Science Foundation awards (EF-0949047 and 1048199), and XSEDE allocation (DEB100001) on the Texas Advanced Computing Center Ranger. The funders had no role in the study design, data collection and analysis, decision to publish or preparation of the manuscript.

*Conflict of Interest*: none declared.
